# The use of emergency department and outpatient clinics by Syrian refugees

**DOI:** 10.7189/jogh.09.020404

**Published:** 2019-12

**Authors:** Necmi Baykan, Mehmet Ali Aslaner

**Affiliations:** Nevşehir State Hospital, Emergency Department, Nevşehir, Turkey

## Abstract

**Background:**

Displacement after a war or an armed conflict always leads to unexpected health problems, both among migrating people and in places to which new people have migrated. This study aimed to determine the health care needs and trends of Syrian patients.

**Methods:**

This retrospective study was conducted in a secondary care hospital in the city of Nevşehir, in central Turkey, between January 2013 and December 2017. All Syrian patients who visited the outpatient clinics and emergency department (ED) were enrolled in the study.

**Results:**

Over a span of five years, 41 723 Syrian patients visited the hospital’s outpatient clinics and ED. The patients’ median age was 23 (inter-quartile range (IQR) = 7-34), and 57.7% of them were female. In 2017, one-third of the Syrian patients visited the ED, a rate that was higher than that found among local patients (30.3% vs 25.0%, *P* < 0.001, respectively). The rate of pediatric clinic admissions among Syrian patients was about four times greater than the rate of local patients (20.1% vs 5.2%, *P* < 0.001, respectively), and Syrians’ rate of admission to the obstetrics and gynecology clinic was about three times greater than the rate of local patients’ admissions (12.3% vs 4.3%, *P* < 0.001, respectively).

**Conclusions:**

This study showed that Syrian patients’ visits to the hospital, and especially the ED, are increasing. Further, the needs and expectations of these patients in terms of health care are different from local demands. New approaches should be applied to provide an appropriate use of health care facilities.

War is a public health problem that leads to the destruction of nature and humankind; it is a threat to social life, and world leaders have agreed that there is a global refugee and migration crisis today stemming from battles, armed conflicts, and human rights violations in many parts of the world [1]. When it comes to migration, those countries that border states without conflict as well as overseas countries, are becoming an option for people fleeing undesirable locations.

Turkey has been one of the countries with the highest rates of immigration arrivals since the start of the Syrian civil war in 2011. Officially, more than 3.5 million Syrians were recorded by the Disaster and Emergency Management Authority as being in Turkey as of April 2018 [2]. This number accounts for roughly 4.4% of Turkey’s population. In Nevşehir, where the study was conducted, the number of Syrian refugees in 2017 was reported by the Directorate General for Migration Management at 7.819; this accounts for 2.69% of the province’s population [3].

Healthcare is an important challenge for Syrian refugees, due to the language barrier, differences in cultural background, and the physical and psychological disturbances they face after trauma [4]. Further, there is not enough data regarding in which health care areas they need care and which outpatient or emergent care clinics they prefer when they visit the hospital. This study aims to determine which clinics are preferred by Syrian patients and to compare this data to the use of these clinics by other local patients.

## METHODS

### Study design

This retrospective study was conducted in a secondary care hospital in Nevşehir, central Turkey, between January 2013 and December 2017. Few data were available for analysis prior to 2013; therefore, the data were collected beginning with that year. All patients (Syrian and local, separately) who visited an outpatient clinic or the emergency department (ED) were enrolled in the study. Informed consent form was not asked for due to study design. Nevşehir Hacı Bektaş University Ethics Committee approved the study (2018.05.62). The study protocol was previously published on ClinicalTrials.gov (NCT03040843).

The hospital is the city’s largest public health care facility, and it has the most admissions in the city. All Syrian refugees’ health care payments are paid by the government health insurance system, and health insurance is valid only in the city where a person is registered as living; therefore, nearly all of the patients who are registered in Nevşehir visit this public hospital when they are in need of health care.

### Data collection

Data on Syrian refugees and other patients were obtained from the hospital’s medical records. Demographic characteristics, first diagnoses upon admission, type of clinic visited, and the patients’ final status were recorded. The *International Statistical Classification of Diseases and Related Health Problems* 10^th^ Revision (ICD-10) was used to define patients’ first diagnoses. In cases of missing data, the patients were excluded from the study.

### Data analysis

Statistical analyses were performed using IBM SPSS Statistics for Windows, Version 21 (IBM, Armonk, NY, USA). Continuous variables were presented as median values and interquartile ranges (IQRs). Categorical variables were summarized as frequencies and percentages. Categorical variables were compared using Pearson χ2 or Fisher exact test. A critical α value of .05 was accepted as statistically significant.

## RESULTS

Between 2013 and 2017, 41 723 hospital visits were made by Syrian patients, including outpatient clinics and ED. The patients’ median age was 23 (IQR = 7-34), and 57.7% of them were female. During the study period, 52.2% of all Syrian hospital visits occurred in 2017, 32.6% in 2016, 11.1% in 2015, 3.8% in 2014, and 0.3% in 2013.

Symptoms, signs, and abnormal clinical and laboratory findings (ICD-10, R00-R99) were the most common diagnostic codes, at 18.1% (which were 66.4% pain related codes, 8.2% nausea-vomiting, 3.9% fever, and others), followed by diseases of the respiratory system (ICD-10, J00-J99) at 17.0% (which were 43.4% upper respiratory tract [non-infection] related codes, 42% upper respiratory tract infection, 11.8% lower respiratory tract infection, and others). Factors influencing health status (ICD-10, Z00-Z99) accounted for 15.1% of diagnostic codes (which were 50.9% general child/adult medical examination, 39% pregnancy related codes, and others), and diseases of the musculoskeletal system and connective tissue (ICD-10, M00-M99) were 10.5%.

The patient’s discharge was the final decision for 91.3% of all Syrian patients, an admission decision accounted for 6.9% of them, and 1.8% were referred to another hospital. Of all these patients, 31.1% visited the ED, and 68.9% visited an outpatient clinic. Of these ED visits, 87.2% resulted in the patient being discharged, 12.2% were admitted, and 0.6% were referred.

In the last five years, the number of Syrian patients in this city has increased exponentially (**Table 1**). This increase outpaced that of other patients. The rate of Syrian to non-Syrian ED visits was higher than the rate of Syrian to non-Syrian outpatient clinic visits as 54.5% for all years. While this rate was 133.3% in 2013, it was only 31.8% in 2017. The rate of ED to clinic visits amongst refugees was higher than that of non-refugee population (36% and 15%, respectively) in 2013. These rates of refugee and non-refugee population were 43% and 33% (respectively) in 2017.

**Table 1:** Numbers of Syrian patients who visited the outpatient clinics and emergency department each year (2013–2017)

		2013	%	2014	%	2015	%	2016	%	2017	%
Outpatient clinics	Syrian patients (n)	92	0.03	1203	0.3	2964	0.5	9292	1.4	15195	2.2
	Other patients (n)	314713		446548		593386		652903		691782	
Emergency department	Syrian patients (n)	33	0.07	392	0.5	1660	0.9	4289	1.9	6603	2.9
	Other patients (n)	47024		80205		184566		220155		230675	

During 2017, the year in which the most hospital admissions were seen, one-third of Syrian patients visited the ED; this rate was higher than that of other patients (30.3% vs 25.0%, *P* < 0.001, respectively) (**Table 2**). The rate of pediatrics admissions among Syrian patients was about four times greater than among other patients (20.1% vs 5.2%, *P* < 0.001, respectively), and Syrians’ rate of obstetrics and gynecology (O&G) admissions was about three times greater than the rate of other patients’ admissions (12.3% vs 4.3%, *P* < 0*.*001, respectively). Diseases of the respiratory system were the most common diagnoses among Syrian patients, accounting for about one-fifth of all diagnoses in 2017 (**Table 2**).

**Table 2:** Characteristics of Syrians and other patients who visited to the outpatient clinics and emergency department in 2017

	Syrian patients, n = 21798	Other patients, n = 922456	*P*-value
**Numbers of admissions (%)**:			
Emergency department	6603 (30.3%)	15195 (25.0%)	<0.001
Pediatrics	4380 (20.1%)	48070 (5.2%)	
Obstetrics and gynecology	2674 (12.3%)	39632 (4.3%)	
Orthopedics	1211 (5.6%)	54860 (5.9%)	
ENT	1077 (4.9%)	63258 (6.9%)	
Internal medicine	851 (3.9%)	75053 (8.1%)	
Dermatology	711 (3.3%)	23030 (2.5%)	
Ophthalmology	700 (3.2%)	48249 (5.2%)	
General surgery	418 (1.9%)	32325 (3.5%)	
Other	3173 (14.6%)	307305 (33.3%)	
**Numbers of admission diagnoses (%)** [5]:			
Diseases of the respiratory system	4239 (19.4%)	139830 (15.2%)	<0.001
Factors influencing health status	3407 (15.6%)	118797 (12.9%)	
Symptoms, signs, and abnormal clinical and laboratory findings	3056 (14.0%)	116820 (12.7%)	
Diseases of the musculoskeletal system and connective tissue	2366 (10.9%)	124798 (13.5%)	
Diseases of the genitourinary system	1381 (6.3%)	54087 (5.9%)	
Diseases of the digestive system	1028 (4.7%)	40054 (4.3%)	
Injury, poisoning, and certain other consequences of external causes	876 (4.0%)	29963 (3.2%)	
Diseases of the skin and subcutaneous tissue	838 (3.8%)	26521 (2.9%)	
Diseases of the eye and adnexa	738 (3.4%)	49394 (5.4%)	
Other	3869 (17.7%)	222192 (24.1%)	

ENT – ear, nose, and throat

## DISCUSSION

The right to health is considered one of the main issues of human rights, and migration has become one of the most important public health issues for refugees in modern Turkey due to the expected problems of migration such as the language barrier, difficulties in obtaining identity card due to too much applicants at the beginning, living in shelters for a certain period of time, lack of awareness of their rights, and lack of knowledge of the Turkish health care system [6,7]. During a five-year period, we found that more than 40 000 hospital visits were made by Syrian patients in Nevşehir, Turkey. By and large, these patients were young (median age 23) and female. Although the gender ratio varies across studies, the relatively young median age is similar, as its range is roughly 19-30 [4,8].

Since 2013, we found that the number of Syrian patients in Nevşehir has risen dramatically. Whereas there were only 125 Syrian patient visits in 2013, the number grew to 21 798 in 2017; more than 50% of total visits occurred in the last year of the study. A study by Gulacti and colleagues in Turkey reported that ED visits increased by only 6.5% during five years (2010-2015) by Syrian patients [9]. This rate is very low compared to our results. This discrepancy may be related to where Gulacti et al.’s study was conducted. Their work described an area very close to the Turkey-Syria border, and a number of Syrian refugees have lived there for a long time. By contrast, Nevşehir is located in central Turkey, so Syrian refugees’ migration has been increasing in this region over time. While the number of registered Syrian refugees accounted for 0.06% of this city population in 2014, this rate increased 2.69% in 2017 [3,10].

When we examined the outcomes of the Syrian patients’ visits, we found that the majority of them were discharged from the ED; this rate was similar to that reported in previous studies [9,11]. Another study conducted in Turkey noted that inappropriate ED use by Syrian patients was very high, about 68% [9]. In addition, roughly one-third of refugee patients use EDs for general health care needs [4,8]. In our study, we found that ED use by Syrian patients was higher than that of local patients, and the majority of these patients actually needed only outpatient care. The most likely causes of this situation are the easy access to the ED, less language barrier due to less formal procedures in the ED and limited working hours associated with outpatient clinics [9,11].

In this study, pediatrics visits by Syrian patients were four times greater, and O&G visits were three times more frequent, than those by local patients. We know that there are nearly 20 million refugees worldwide, and more than half of them are children [12]. All studies regarding refugee patients in Turkey also reported these patients’ relatively young ages. Aside from being outnumbered, these children are vulnerable to nutritional deficiencies, infectious diseases, and mental health disorders [6]. Increased deliveries among refugees over the local population is another important situation that can be mostly related to their low socioeconomic status, poverty, forced marriage, reduced use of modern contraceptives, and unwanted pregnancies [13-15]. Each of these factors is a likely cause for the increased need for health care in these areas among refugee patients.

Suggestions:

- Firstly, Syrians living conditions should be improved regarding preventive health care for children, women who are reproductive age and vulnerable groups.
- Syrian refugees need to be educated and referred to the primary health care services for simple complaints in order to reduce the rates of admissions to hospitals.
- Comprehensive reproductive health services should be integrated into primary health care in order to prevent unplanned or forced pregnancies.
- Language resources need to be put in, especially, primary health care services and other services until language learning process is completing.
- Home Health Care Services are used in many cities in Turkey. This care can also be extended for refugees.
- Finally, national and local health goverments need to increase support for EDs, pediatrics, and O&G clinics due to increased demand from refugees.

## CONCLUSIONS

As a result of rapidly increasing number of refugees, the demands, needs, and expectations for health care in Turkey are changing. The present study draws attention to these patients’ tendency to visit the ED and other, specific clinics, such as pediatrics and obstetrics and gynecology, compared to the native Turkish population, although the majority of these patients actually needed only outpatient care. Providing quality health care in primary health care settings, extending preventive health care, and taking precautions regarding language problems would make care more accessible and easier to provide, while also reducing unnecessary visits by these patients to the hospital.
